# Risk factors for progression to severe infection and prolonged viral clearance time in hospitalized elderly patients infected with the Omicron variant of SARS-CoV-2: a retrospective study at Shanghai Fourth People's Hospital, School of Medicine, Tongji University

**DOI:** 10.3389/fmicb.2024.1361197

**Published:** 2024-04-15

**Authors:** Siqi Tang, Qiuhong Man, Dongliang Zhu, Xueying Yu, Ruilin Chen, Shuo Wang, Yihan Lu, Qiqing Shi, Chen Suo, Lize Xiong

**Affiliations:** ^1^Shanghai Institute of Infectious Disease and Biosecurity, Fudan University, Shanghai, China; ^2^Department of Epidemiology, Ministry of Education, Key Laboratory of Public Health Safety, School of Public Health, Fudan University, Shanghai, China; ^3^Department of Clinical Laboratory, Shanghai Fourth People's Hospital, School of Medicine, Tongji University, Shanghai, China; ^4^Department of Anesthesiology and Perioperative Medicine, Shanghai Fourth People's Hospital, School of Medicine, Tongji University, Shanghai, China; ^5^Shanghai Key Laboratory of Anesthesiology and Brain Functional Modulation, Translational Research Institute of Brain and Brain-Like Intelligence, Shanghai Fourth People's Hospital, School of Medicine, Tongji University, Shanghai, China

**Keywords:** SARS-CoV-2, Omicron variant, COVID-19, risk factor, elderly

## Abstract

**Introduction:**

In elderly patients infected with the Omicron variant, disease progression to severe infection can result in poor outcomes. This study aimed to identify risk and protective factors associated with disease progression to severe infection and viral clearance time in elderly Omicron-infected patients.

**Methods:**

Shanghai Fourth People's Hospital, School of Medicine, Tongji University, was officially designated to provide treatment to patients with COVID-19. This study was conducted on confirmed Omicron cases admitted to the hospital between 10 April 2022 and 21 June 2022. In total, 1,568 patients aged 65 years or older were included. We conducted a retrospective, observational study using logistic regression to analyze risk and protective factors for the development of severe disease and Cox proportional hazards regression models to analyze factors influencing viral clearance time.

**Results:**

Aged over 80 years, having 2 or more comorbidities, combined cerebrovascular disease, chronic neurological disease, and mental disorders were associated with the development of severe disease, and full vaccination was a protective factor. Furthermore, aged over 80 years, combined chronic respiratory disease, chronic renal disease, cerebrovascular disease, mental disorders, and high viral load were associated with prolonged viral clearance time, and full vaccination was a protective factor.

**Discussion:**

This study analyzed risk factors for progression to severe infection and prolonged viral clearance time in hospitalized elderly Omicron-infected patients. Aged patients with comorbidities had a higher risk of developing severe infection and had longer viral clearance, while vaccination protected them against the Omicron infection.

## Background

Since its first discovery in South Africa in November 2021, the Omicron variant of severe acute respiratory syndrome coronavirus 2 (SARS-CoV-2) has caused pandemics worldwide due to its strong infectiousness and variability, including subvariants of BA.1, BA.2, BA.3, BA.4, BA.5, and BF.7 (Saito et al., 2022; Viana et al., 2022; Wolter et al., 2022). The two epidemics of Omicron strains (BA.2.2, BA.5, and BF.7) in Shanghai, China caused a rapid spread among the population, resulting in a large number of elderly patients requiring hospitalization after being infected (Chen Z. et al., 2022). Early identification and warning signs for patients with underlying high-risk potential Omicron severe infections are key to clinical management to reduce the incidence of severe infection. Although previous studies have explored the risk and protective factors associated with disease progression to severe disease in COVID-19-infected patients (Tehrani et al., 2021; Sacco et al., 2022; Solis et al., 2022), the Omicron lineage could present different clinical features in a virtually infection-naive population (Harvey et al., 2021; Carabelli et al., 2023). In particular, older populations demonstrated increased susceptibility and risk factors compounded by significantly lower vaccination uptake among this demographic, exacerbating their vulnerability (Wang et al., 2023). Few previous prognostic models have anticipated the epidemiological and clinical characteristics of the Omicron variant alone for the vulnerable group of elderly patients with comorbidities, while these models were also designed for the Omicron elderly with severe infection.

Therefore, our research was focused on identifying the risk and protective factors that influence the transition to severe infection and the time required for viral clearance in elderly patients infected with the Omicron variant. Utilizing the epidemiological and clinical characteristics of Omicron infections, we aimed to accurately predict the progression of the disease in this demographic of elderly population. This predictive capability is intended to facilitate the formulation and implementation of combined clinical and preventive interventions aimed at reducing the incidence of severe manifestations among those infected with the Omicron variant.

## Methods

### Study design

This study was conducted on confirmed Omicron cases admitted to Shanghai Fourth People's Hospital, School of Medicine, Tongji University, which was officially designated to provide treatment to patients with COVID-19, between 10 April 2022, and 21 June 2022. We retrospectively analyzed the epidemiological and clinical data of 1,568 Omicron elderly patients aged > 65 years during the Omicron BA.2 epidemic in Shanghai to uncover the risk factors associated with the occurrence of serious illness and the prolongation of viral clearance time in Omicron infections and to build a prediction model.

The inclusion criteria for the study population are as follows: (1) aged ≥65 years and (2) positive for SARS-CoV-2 by real-time polymerase reaction chain (RT-PCR) or hospitalized with a diagnosis related to COVID-19 (ICD-10: U071, U072, and U073). We further excluded patients with more than 30% of the covariate information missing. For missing values in continuous variables, we employ mean imputation; for categorical variables, mode imputation is used. Patients meeting one of the following conditions were identified as severe cases according to the Diagnosis and Treatment Scheme of Pneumonia Caused by Novel Coronavirus of China (the ninth version): (1) the presence of shortness of breath with RR ≥ 30 breaths/min; (2) oxygen saturation ≤ 93% in the resting state while breathing room air; (3) arterial partial pressure of oxygen (PaO2)/inhaled oxygen concentration (FiO2) ≤ 300 mmHg; (4) progressive worsening of clinical symptoms, with lung imaging showing the significant progression of >50% of the lesion within 24–48 h; (5) respiratory failure and the need for mechanical ventilation; (6) shock; and (7) combined with other organ failure requiring ICU monitoring treatment. We excluded patients with a diagnosis of severe disease at admission to explore the influencing factors that play a role in its progression.

### General characteristics

We obtained epidemiological information on the general demographic characteristics and diseases suffered before being infected with the Omicron virus for each study subject from the clinical electronic medical record system of the Shanghai Fourth People's Hospital. The vaccination status was also included, and the population was divided into two groups based on the number of vaccinations: full vaccination (2 or more doses) and no full vaccination (0 or 1 dose).

The main types of comorbidities included in this study in elderly Omicron-infected patients are as follows: hypertension, diabetes, heart disease, cerebrovascular disease (cerebral infarction, cerebral hemorrhage, etc.), chronic respiratory disease (chronic obstructive pulmonary disease, chronic bronchitis, emphysema, etc.), chronic nephrotic disease (nephrotic syndrome, renal insufficiency, renal failure, etc.), chronic neurological diseases (Parkinson's disease, epilepsy, Alzheimer's disease, senile brain atrophy, etc.), and mental disorders (schizophrenia, dementia, depression, etc.). Other diseases included diseases of the blood and blood-forming organs, autoimmune diseases, trauma with fractures, and organ transplantation status.

### Laboratory indicators

In this study, the Ct value of RT-PCR was used as an important index to monitor the viral shedding time during the treatment of elderly Omicron-infected patients. Nasal and pharyngeal swabs were collected daily from the patients, and RT-PCR was performed to record the changes in the Ct value.

The study involved the use of routine blood tests to detect disease progression. These tests include white blood cell count, neutrophil count, lymphocyte count, monocyte count, eosinophil count, basophil count, interleukin-6, hemoglobin, and D-dimer within 24 h of the first day of hospitalization.

### Statistical analysis

We analyzed the epidemiological baseline characteristics of Omicron elderly patients with severe infection and non-severe infection using mean (±standard deviation) if they were Gaussian distributions by the Kolmogorov–Smirnov test. Otherwise, they were expressed as medians and interquartile ranges. A *t*-test and a chi-squared test were used to compare the differences in each factor between the two groups. Univariate and multivariate logistic regression was used to analyze the effect of general demographic characteristics, comorbidities, and vaccination on whether Omicron-infected elderly patients developed severe disease. Viral clearance was determined by conducting two consecutive negative RT-PCR tests (CT > 35) after a positive nucleic acid test with an interval of >24 h, according to the *Diagnosis and Treatment Scheme of Pneumonia Caused by Novel Coronavirus of China* (the ninth version). Viral shedding time was defined as the time interval between the first positive nucleic acid test and the first negative test in viral clearance. We used Cox proportional hazards models to analyze the effect of the Ct value of the nucleic acid in the first admission test on the time to viral clearance of patients, adjusting by age, sex, and comorbidities. Finally, we divided all cases into training and validation sets using a 10-fold cross-validation method. In the training set, we included variables such as general demographic characteristics of patients, different types of comorbidities, vaccination status, and Ct values of RT-PCR at admission to construct a prediction model, and in the validation set, we validated the model to predict the risk of patients developing severe disease. The model was validated in the validation set to predict the risk of patients developing severe disease, and the model effects were evaluated. All analyses were performed using R 4.2.0, and *p*-values of <0.05 (two-sided test) were considered statistically significant.

## Results

### Epidemiological and clinical characteristics of elderly patients with severe and non-severe Omicron infections

According to the inclusion criteria, a total of 1,568 elderly patients with Omicron infection were finally included in our study, with a median age of 81.0 (IQR: 72.4, 88.3) years. Of these patients, there were 640 men (40.8%) and 928 women (59.2%); among the included elderly patients, 215 (13.2%) were severe cases, with a median age of 86.0 (IQR: 76.4, 90.5) years, comprising 95 men (44.2%) and 120 women (55.8%). The remaining 1,353 cases were non-serious (86.8%), with a median age of 79.9 (IQR: 72.0, 87.7) years, consisting of 545 men (40.3%) and 425 women (59.7%). The majority of Omicron elderly patients had comorbidities (84.6%); more than half of the elderly patients had two or more types of comorbidities (53.7%); and the vast majority of elderly patients with infection were not fully vaccinated (89.8%). The median length of hospital stay was 10 days, and the median viral shedding time was 8 days.

The epidemiological characteristics were significantly different between severe and non-severe cases, with a greater proportion of cases progressing to severe infection in the advanced age group than non-severe cases (*P* < 0.001); patients progressing to severe disease had a greater proportion of comorbidities, with more than two comorbidities being significantly associated with sever disease (*P* < 0.001).

Patients who received the full vaccine were less likely to develop severe disease (*P* < 0.001). The length of hospital stays and the viral shedding time in the severe group were longer than those in the non-severe group (*P* < 0.001).

The O-gene (*P* = 0.001) and N-gene (*P* = 0.001) Ct values were lower in the severe group and the white blood cell count (*P* < 0.001), neutrophil count (*P* < 0.001), interleukin-6 (*P* < 0.001), and D-dimer (*P* < 0.001) levels were higher in the same group, while the lymphocyte count (*P* = 0.008), basophil count (*P* < 0.001), and hemoglobin (*P* = 0.005) levels were lower in the severe group. Comorbidities such as heart disease (*P* = 0.002), cerebrovascular disease (*P* < 0.001), chronic neurological disease (*P* < 0.001), and psychiatric disorders (*P* < 0.001) were more common in patients with severe Omicron infection.

There were statistically significant differences in the provision of respiratory support, including oxygen therapy (*P* < 0.001), non-invasive mechanical ventilation (*P* < 0.001), and endotracheal intubation (*P* < 0.001), between those who progressed to severe illness and those who remained non-severe. The incidence of requiring such interventions was notably higher among the severe cases (Table 1).

**Table 1:** Epidemiological and clinical characteristics of elderly patients with severe and non-severe Omicron infections.

	**Total**	**Non-severe**	**Severe**	***P*-value**
	**(*N* = 1,568)**	**(*N* = 1,353)**	**(*N* = 215)**	
Sex, male (%)	640 (40.8)	545 (40.3)	95 (44.2)	0.314
Age, median (IQR)	81.0 (72.4, 88.3)	79.9 (72.0, 87.7)	86.0 (76.4, 90.5)	**< 0.001**
Length of stay, day (IQR)	10.0 (6.0, 14.0)	10.0 (6.0, 14.0)	13.0 (9.0, 18.0)	**< 0.001**
Viral shedding time, day (IQR)	8.00 (4, 12.0)	8.0 (4.0, 12.0)	11.0 (7.0, 16.0)	**< 0.001**
**Comorbidity**	1,239 (82.1)	1,068 (81.6)	171 (85.1)	0.271
< 2 types	700 (46.3)	635 (48.4)	65 (32.3)	**< 0.001**
≥2 types	812 (53.7)	676 (51.6)	136 (67.7)	
Heart disease	458 (30.3)	378 (28.9)	80 (39.8)	**0.002**
Hypertension	911 (60.3)	786 (60.0)	125 (62.2)	0.616
Chronic respiratory disease	132 (8.7)	109 (8.3)	23 (11.4)	0.186
Cancer	60 (4.0)	54 (4.1)	6 (3.0)	0.564
Diabetes	368 (24.4)	325 (24.8)	43 (21.4)	0.333
Chronic kidney disease	73 (4.8)	59 (4.5)	14 (7.0)	0.182
Cerebrovascular disease	348 (23.0)	274 (20.9)	74 (36.8)	**< 0.001**
Chronic nervous disease	103 (6.8)	73 (5.6)	30 (14.9)	**< 0.001**
Mental disorder	213 (14.1)	158 (12.1)	55 (27.4)	**< 0.001**
**Vaccination**				
Partly vaccinated/not vaccinated	1,408 (89.8)	1,199 (88.6)	209 (97.2)	**< 0.001**
Fully vaccinated	160 (10.2)	154 (11.4)	6 (2.8)	
**RT-PCR results**				
Combined Ct value				0.056
≤ 20^a^	389 (26.4)	323 (25.4)	66 (32.0)	
>20^b^	1,087 (73.6)	947 (74.6)	140 (68.0)	
**Laboratory examinations**				
White blood cell count, × 10^9^ (SD)	5.83 (2.93)	5.60 (2.59)	7.28 (4.23)	**< 0.001**
Neutrophil count, × 10^9^ (SD)	3.90 (2.64)	3.65 (2.22)	5.52 (4.11)	**< 0.001**
Lymphocyte count, × 10^9^ (SD)	1.37 (1.15)	1.40 (1.20)	1.17 (0.72)	**0.008**
Monocyte count, × 10^9^ (SD)	0.47 (0.23)	0.47 (0.21)	0.50 (0.36)	0.056
Eosinophil count, × 10^9^ (SD)	0.07 (0.11)	0.07 (0.10)	0.06 (0.13)	0.141
Basophil count, × 10^9^ (SD)	0.02 (0.01)	0.02 (0.01)	0.01 (0.01)	**< 0.001**
Platelet count, × 10^9^ (SD)	188.88 (76.80)	188.89 (77.14)	188.82 (74.16)	0.993
Interleukin-6 (IQR)	32.2 (15.2, 117.5)	30.2 (13.9, 107.8)	47.0 (23.2, 157.6)	**< 0.001**
Hemoglobin (SD)	122.6 (19.0)	123.1 (18.6)	119.2 (20.8)	**0.005**
D-dimer (IQR)	0.64 (0.38, 1.30)	0.60 (0.37, 1.16)	1.30 (0.54, 2.70)	**< 0.001**
**Respiratory support**				
Oxygen therapy	40 (2.6)	2 (0.1)	38 (17.7)	**< 0.001**
Non-invasive mechanical ventilation	98 (6.3)	10 (0.7)	88 (40.9)	**< 0.001**
Endotracheal intubation	42 (2.7)	2 (0.1)	40 (18.6)	**< 0.001**

a Either O-gene Ct values or N-gene Ct values were ≤ 20.

b Both O-gene Ct values and N-gene Ct values were >20. Bold indicates *P* < 0.05.

### Analysis of risk factors associated with Omicron elderly patients with severe infections

According to the univariate logistic regression analysis, it could be found that age > 80 years, having at least two comorbidities, and an O-N gene Ct value ≤ 20 on admission testing were the risk factors for severe disease in elderly patients with Omicron infection, and a high white blood cell count, a high neutrophil count, and a high interleukin-6 might suggest an increased risk of developing severe disease. Among them, the risk was highest for those aged > 80 years (cOR = 2.22, 95% CI 1.64–3.03). Among the comorbidities, heart disease, cerebrovascular disease, and mental disorders were the risk factors. Full vaccination was a protective factor for severe infection (cOR = 0.22, 95% CI 0.09–0.47).

Further multivariate logistic regression analysis was performed to clarify the confounding effects of the risk factors. The effects of each influential factor were adjusted by sex, age, comorbidities, and vaccination status in the model. The results found that the following factors were associated with an increased risk of severe disease: being male (aOR = 1.56, 95% CI 1.13–2.14), being 80 years or older (aOR = 1.82, 95% CI 1.31–2.57), having a combination of two or more comorbidities (aOR = 1.63, 95% CI 1.19–2.26), and having an admission nucleic acid test Ct value ≤ 20 (aOR = 1.40, 95% CI 1.00–1.94), and full vaccination were significantly and negatively associated with severe disease (Table 2).

**Table 2:** Risk factors for the development of severe disease in elderly Omicron-infected patients.

	**Univariate regression analyses**		**Multivariate regression analyses**	
	**Crude OR (95% CI)**	***P*-value**	**Adjusted OR (95% CI)**	***P*-value**
**Sex**				
Female	Ref		Ref	
Male	1.17 (0.88, 1.57)	0.279	**1.56 (1.13, 2.14)**	0.006^a^
**Age (years)**				
65–80	Ref		Ref	
≥80	**2.22 (1.64, 3.03)**	< 0.001	**1.82 (1.31, 2.57)**	< 0.001^b^
**Comorbidity**				
≥2 types	**1.75 (1.29, 2.38)**	< 0.001	**1.63 (1.19, 2.26)**	0.003^c^
Heart disease	**1.63 (1.20, 2.21)**	0.002	1.18 (0.83, 1.67)	0.357^c^
Hypertension	1.1 (0.81, 1.49)	0.560	0.74 (0.52, 1.06)	0.101^c^
Chronic respiratory disease	1.42 (0.87, 2.26)	0.145	1.16 (0.69, 1.86)	0.558^c^
Cancer	0.72 (0.27, 1.56)	0.445	0.72 (0.27, 1.6)	0.460^c^
Diabetes	0.82 (0.57, 1.17)	0.286	**0.58 (0.39, 0.85)**	0.006^c^
Chronic kidney disease	1.59 (0.84, 2.82)	0.132	1.29 (0.67, 2.33)	0.427^c^
Cerebrovascular disease	**2.21 (1.60, 3.02)**	< 0.001	**1.67 (1.17, 2.38)**	0.005^c^
Chronic nervous disease	**2.98 (1.87, 4.64)**	< 0.001	**2.41 (1.49, 3.82)**	< 0.001^c^
Mental disorder	**2.75 (1.92, 3.89)**	< 0.001	**2.44 (1.68, 3.53)**	< 0.001 ^c^
**Vaccination**				
Partly vaccinated/not vaccinated	Ref		Ref	
Fully vaccinated	**0.22 (0.09, 0.47)**	< 0.001	**0.27 (0.09, 0.61)**	0.005^d^
**RT-PCR results**				
Combined Ct value				
>20	Ref		Ref	
≤ 20	**1.38 (1.00, 1.89)**	0.046	**1.40 (1.00, 1.94)**	0.048 ^e^
**Laboratory examinations**				
White blood cell count (per SD)	**1.56 (1.37, 1.78)**	< 0.001	**1.61 (1.40, 1.86)**	< 0.001^e^
Neutrophil count (per SD)	**1.73 (1.52, 1.97)**	< 0.001	**1.78 (1.54, 2.06)**	< 0.001^e^
Lymphocyte count (per SD)	**0.56 (0.41, 0.75)**	< 0.001	**0.67 (0.49, 0.91)**	0.015^e^
Monocyte count (per SD)	1.13 (0.99, 1.28)	0.064	1.10 (0.96, 1.25)	0.155^e^
Eosinophil count (per SD)	0.87 (0.72, 1.03)	0.142	0.98 (0.81, 1.14)	0.774^e^
Basophil count (per SD)	**0.60 (0.51, 0.71)**	< 0.001	**0.65 (0.55, 0.77)**	< 0.001^e^
Platelet count (per SD)	0.88 (0.59–1.27)	0.500	1.02 (0.7–1.47)	0.898^e^
Interleukin-6 (per SD)	**1.01 (1.00, 1.02)**	< 0.001	**1.01 (1.00, 1.02)**	0.013^e^
Hemoglobin (per SD)	**0.99 (0.98, 1.00)**	0.005	0.99 (0.99, 1.00)	0.087^e^
D-dimer (per SD)	1.01 (1.00, 1.03)	0.079	1.01 (0.99, 1.03)	0.180^e^

Logistic regression models were used.

a Adjusted by age, comorbidity, and vaccination in the multifactorial model.

b Adjusted by sex, comorbidity, and vaccination.

c Adjusted by sex, age, the presence of more than two comorbidities, and vaccination.

d Adjusted by sex, age, and comorbidities.

e Adjusted by sex, age, comorbidities, and vaccination. Bold indicates *P* < 0.05.

### Analysis of factors influencing viral shedding time in elderly Omicron-infected patients

In the analysis of viral shedding time, we applied a Cox regression model. We found that age ≥80 years (aHR = 0.75, 95% CI 0.67–0.84), admission test with an O-N gene Ct value ≤ 20 (aHR = 0.65, 95% CI 0.55, 0.76), and having a chronic respiratory disease (aHR = 0.79, 95% CI 0.66–0.95), chronic kidney disease (aHR = 1.45, 95% CI 1.13–1.86), cerebrovascular disease (aHR = 0.86, 95% CI 0.75–0.98), or mental disorder (aHR = 0.84, 95% CI 0.72–0.98) would prolong the time to viral clearance and be detrimental to patient prognosis. In contrast, full vaccination (1.44, 1.21, and 1.72) was beneficial in reducing the time to viral clearance. A high white blood cell count, neutrophil count, eosinophil count, basophil count, and platelet count at admission suggested a shortened time to viral clearance (Table 3).

**Table 3:** Analysis of factors influencing the viral shedding time in elderly Omicron-infected patients.

	**Univariate regression analyses**		**Multivariate regression analyses**	
	**Crude HR (95% CI)**	***P*-value**	**Adjusted HR (95% CI)**	***P*-value**
**Sex**				
Female	Ref		Ref	
Male	1.02 (0.93, 1.14)	0.636	0.93 (0.83, 1.04)	0.191^a^
**Age (years)**				
65–80	Ref		Ref	
≥80	**0.66 (0.59, 0.73)**	< 0.001	**0.75 (0.67, 0.84)**	< 0.001^b^
**Comorbidity**				
≥2 types	**0.86 (0.78, 0.96)**	< 0.001	0.93 (0.83, 1.03)	0.164^c^
Heart disease	**0.87 (0.78–0.97)**	0.011	0.99 (0.86–1.12)	0.827^c^
Hypertension	0.98 (0.89–1.09)	0.719	1.12 (0.99–1.26)	0.063^c^
Chronic respiratory disease	**0.77 (0.64–0.92)**	0.004	**0.79 (0.66–0.95)**	0.012^c^
Cancer	1.08 (0.84–1.4)	0.542	1.01 (0.77–1.32)	0.935^c^
Diabetes	1.06 (0.94–1.19)	0.332	1.03 (0.9–1.18)	0.655^c^
Chronic kidney disease	**1.32 (1.04–1.67)**	0.021	**1.45 (1.13–1.86)**	0.004^c^
Cerebrovascular disease	**0.77 (0.68–0.87)**	< 0.001	**0.86 (0.75–0.98)**	0.028^c^
Chronic nervous disease	0.87 (0.71–1.06)	0.166	0.93 (0.75–1.14)	0.471^c^
Mental disorder	**0.82 (0.71–0.95)**	0.007	**0.84 (0.72–0.98)**	0.029^c^
**Vaccination**				
Partly vaccinated/not vaccinated	Ref		Ref	
Fully vaccinated	**1.67 (1.42, 1.98)**	< 0.001	**1.44 (1.21, 1.72)**	< 0.001^d^
**RT-PCR results**				
Combined Ct value				
>20	Ref		Ref	
≤ 20	**0.62 (0.54, 0.72)**	< 0.001	**0.65 (0.55, 0.76)**	< 0.001^e^
**Laboratory examinations**				
White blood cell count (per SD)	**1.08 (1.03, 1.13)**	< 0.001	**1.06 (1.01, 1.11)**	0.018^e^
Neutrophil count (per SD)	**1.08 (1.03, 1.14)**	0.003	**1.10 (1.04, 1.16)**	0.001^e^
Lymphocyte count (per SD)	1.02 (0.99, 1.05)	0.263	0.99 (0.96, 1.03)	0.701^e^
Monocyte count (per SD)	1.01 (0.96, 1.06)	0.765	1.03 (0.97, 1.08)	0.348^e^
Eosinophil count (per SD)	**1.14 (1.10, 1.19)**	< 0.001	**1.13 (1.08, 1.19)**	< 0.001^e^
Basophil count (per SD)	**1.17 (1.13, 1.22)**	< 0.001	**1.12 (1.08, 1.17)**	< 0.001^e^
Platelet count (per SD)	**1.53 (1.38–1.71)**	< 0.001	**1.46 (1.3–1.64)**	< 0.001^e^
Interleukin-6 (per SD)	1.00 (1.00, 1.01)	0.063	1.00 (1.00, 1.01)	0.313^e^
Hemoglobin (per SD)	**1.01 (1.00, 1.01)**	0.007	1.00 (0.99, 1.00)	0.412^e^
D-dimer (per SD)	0.99 (0.99, 1.00)	0.083	0.99 (0.98, 1.00)	0.076^e^

Cox proportional hazards models were used.

a Adjusted by age, comorbidity, and vaccination in the multifactorial model.

b Adjusted by sex, comorbidity, and vaccination.

c Adjusted by sex, age, presence of more than two comorbidities, and vaccination.

d Adjusted by sex, age, and comorbidities.

e Adjusted by sex, age, comorbidities, and vaccination. Bold indicates *P* < 0.05.

### Prediction model for elderly Omicron-infected patients toward severe disease

**Figure 1:**
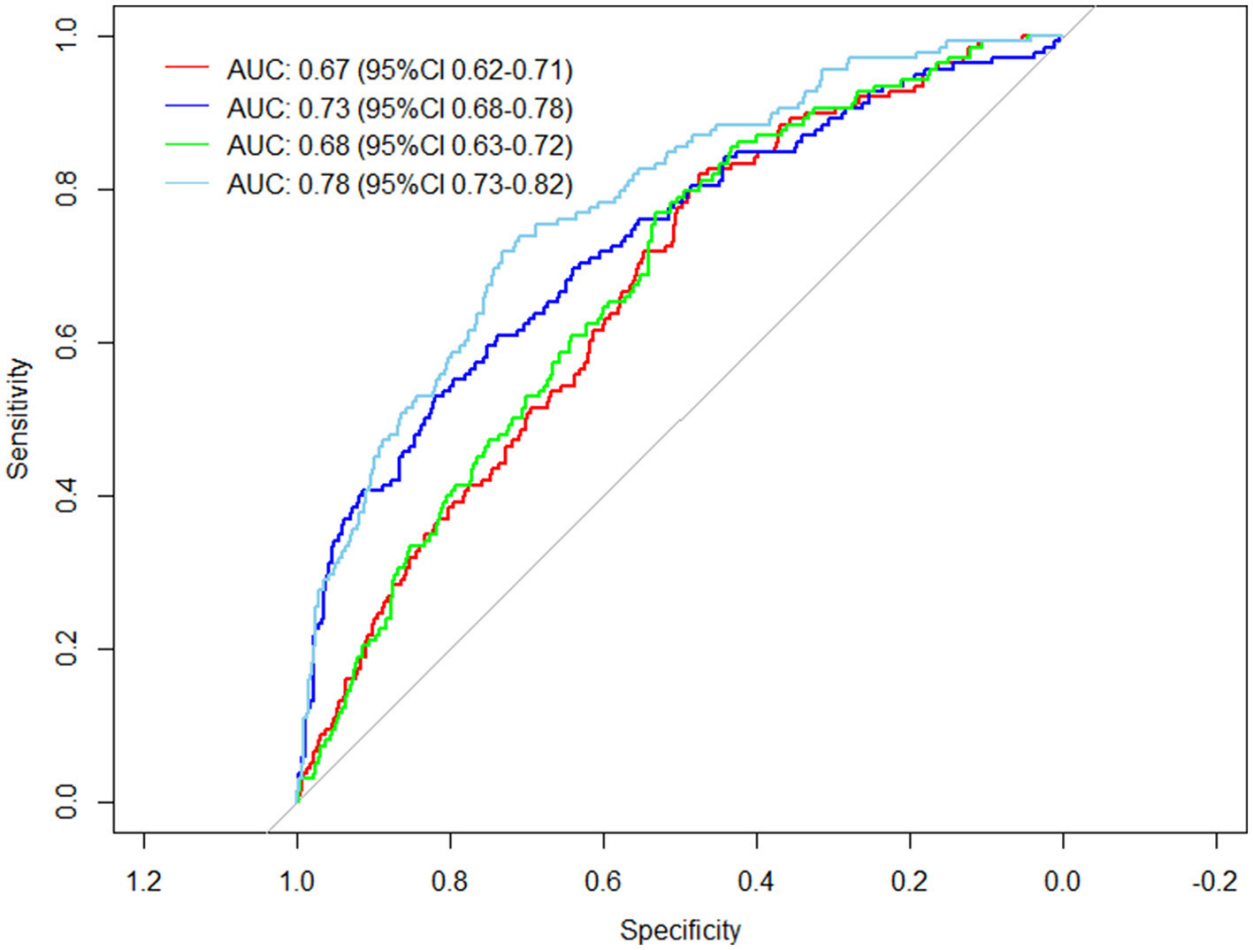
Prediction models for severe illness in elderly Omicron-infected patients.

The general demographic characteristics of patients, comorbidities, vaccination status, and clinical tests were used as predictors to predict the risk of developing severe disease in elderly patients with Omicron infections. A total of four different prediction models were constructed based on the study variables included in the prediction. Model 1 included age, sex, comorbidities, and vaccination status. Model 2 included clinical blood test values: white blood cell count, neutrophil count, lymphocyte count, basophil count, and interleukin-6. Model 3 added the Ct value of the O-N gene at admission as a predictor variable to Model 1. Model 4 included the variables of Model 1 and Model 2 and the Ct value of the O-N gene at admission. It was found that the area under the curve (AUC) was 0.67 (95% CI 0.62–0.71) for Model 1, 0.73 (95% CI 0.68–0.78) for Model 2, 0.68 (95% CI 0.63–0.72) for Model 3, and the best prediction was achieved for Model 4 with an AUC of 0.78 (95% CI 0.73–0.82) (Figure 1).

## Discussion

The Omicron variant, owing to its mutations in the spike protein, demonstrates enhanced transmissibility and a pronounced capacity to circumvent the neutralizing responses elicited by vaccination or previous infection (Abu-Raddad et al., 2022; Willett et al., 2022; Zhang et al., 2022). This trait facilitated its swift dissemination within China, including Shanghai, exerting considerable strain on the healthcare infrastructure. The Omicron variant was the first SARS-CoV-2 strain to achieve widespread transmission among the population of Shanghai, which had limited prior exposure to the virus. It is worth noting that the observed effect in the elderly is possibly due to the vulnerability and immunization status of this population (Chen L. L. et al., 2022). During the epidemic period, early identification of elderly Omicron-infected patients who are at risk of severe disease was crucial to reducing adverse clinical outcomes (Alizadehsani et al., 2021), which might help healthcare workers tailor early intervention strategies, such as more intensive monitoring or prioritization for therapeutic treatments, which could potentially mitigate the severity of the infection. Therefore, this study retrospectively analyzed baseline epidemiological and clinical data of the elderly infected patients during the Omicron pandemic in Shanghai and achieved a model for risk identification by uncovering risk factors leading to adverse clinical outcomes. Early identification of potentially severe patients was important to promote the protection of key populations as well as to offer timely risk stratification management and the rational allocation of medical resources.

Early identification of potentially severe patients was an important way to prevent the development of serious infections and to reduce mortality rates (Kamran et al., 2022). In this study, we found that advanced age (≥80 years), the presence of two or more comorbidities, cerebrovascular disease, chronic neurological disease, and mental disorders were independent risk factors for the development of severe disease in elderly Omicron-infected patients. The inferior outcomes observed in the elderly population can be attributed to a diminished interferon response, which is further exacerbated by the widespread presence of autoantibodies against interferons (Bastard et al., 2020; Cheng and Holland, 2024). The coagulation function of patients with cerebrovascular disease might also be induced by the virus to produce cerebrovascular accidents with serious consequences (De Michele et al., 2022). The association between lung dysfunction and renal disease was already shown (Darmon et al., 2014). Long-term disability due to cerebrovascular disease, chronic neurological disease, and mental disorders might be the reason why this group of patients is more likely to develop severe disease (Tehrani et al., 2021). The study found that fully vaccinated elderly patients were less likely to progress to severe disease. Despite Omicron's immune escape effect, vaccination in the elderly population was an effective way to prevent disease progression to severe disease (Araf et al., 2022; Burki, 2022). The study found diabetes mellitus to be a protective factor for elderly patients. This unexpected finding could be related to the rigorous medical monitoring and self-care practices that diabetic patients often adopt to manage their condition. However, our study did not directly measure lifestyle factors, organ damage, or glycemic control in these patients. We suggest that future studies should incorporate detailed assessments of lifestyle factors, organ damage, and glycemic control to better understand the complex interplay. This approach would provide more insights into whether the observed protective effect is directly related to diabetes mellitus itself, the management of the condition, or other confounding factors.

The viral shedding time is an important determinant of disease transmission and has a large impact on the length of hospitalization (He et al., 2020; Cevik et al., 2021). Therefore, the prediction of viral clearance time in elderly Omicron-infected patients had significant implications for the epidemic prevention policy and the allocation of clinical medical resources. The main risk factors affecting the viral shedding time were found to be advanced age (≥80 years), chronic respiratory disease, chronic renal disease, cerebrovascular disease, mental disorders, and high viral load (Ct < 20), while vaccination was a protective factor.

To quickly identify the risk of serious disease in elderly patients admitted to hospitals with Omicron infection, prediction models were constructed based on the analysis of the related risk factors. The model, which included general demographic characteristics, comorbidities, vaccination status, Ct values, and admission clinical testing indicators, achieved the best result in predicting the incidence of serious illness in elderly patients.

The model we established has some advantages. It focused on elderly patients who were affected during the Omicron epidemic and helped identifying vulnerable individuals promptly. The information was relatively easy to collect at the time of admission or is part of routine clinical examinations and did not require additional training of medical staff or extra examinations, allowing for rapid and effective screening of patients admitted to the hospital (Jain, 2020; Gil et al., 2021; Lu et al., 2022).

This study possesses several limitations that warrant consideration. First, the research was conducted among elderly patients at a single medical center in Shanghai, which restricts its capacity to comprehensively represent the disease spectrum associated with the Omicron variant among the elderly population at large. Consequently, the findings primarily shed light on the clinical outcomes of Omicron infection during the early stages of the outbreak within a population that had limited prior exposure to SARS-CoV-2. This specificity may limit the applicability of our results to broader contexts, particularly in environments where previous infections with SARS-CoV-2 were more prevalent.

Additionally, the study underscores the importance of continued research efforts to update and refine our understanding of the clinical manifestations of COVID-19, particularly in light of changing levels of population immunity and the emergence of new viral variants. Another notable limitation is the absence of comprehensive staging or classification for certain comorbidities, including chronic kidney disease, diabetes mellitus, and cancer. This limitation is acknowledged with the intention that more expansive studies will address these gaps in the future.

The presumption that prior SARS-CoV-2 infections were uncommon among the study participants is based on the stringent “dynamic zero-COVID” policy enforced in China, coupled with the lack of significant outbreaks in Shanghai prior to 2022. Nevertheless, the lack of direct evidence confirming the absence of previous infections represents a limitation. Moreover, the retrospective nature of this study and the constraints on data availability meant that some risk factors were not examined in this analysis.

## Conclusion

The analysis of risk factors and the prediction model for the development of severe disease in elderly patients with Omicron infection contributes to the early detection of high-risk, potentially severe patients and the timely clinical management in risk stratification to prevent patients from progressing to serious infection leading to adverse clinical outcomes.

## Data availability statement

The original contributions presented in the study are included in the article/Supplementary material, further inquiries can be directed to the corresponding authors.

## Ethics statement

The studies involving humans were approved by the Ethics Committee of Shanghai Fourth People's Hospital, School of Medicine, Tongji University (No. 2022099-001) and reported in the Chinese Clinical Trial Register (No. CHiCTR2200065440). The studies were conducted in accordance with the local legislation and institutional requirements. Written informed consent to participate in this study was provided by the participants, their legal guardian or next of kin.

## Author contributions

ST: Formal analysis, Methodology, Software, Writing – original draft. QM: Supervision, Writing – review & editing. DZ: Formal analysis, Methodology, Software, Writing – original draft. XY: Data curation, Writing – review & editing. RC: Data curation, Writing – review & editing. SW: Data curation, Writing – review & editing. YL: Writing – review & editing. QS: Conceptualization, Writing – review & editing. CS: Conceptualization, Investigation, Writing – review & editing. LX: Conceptualization, Writing – review & editing.
