# Supplementary material for: Risk factors for progression to severe infection and prolonged viral clearance time in hospitalized elderly patients infected with the Omicron variant of SARS-CoV-2: a retrospective study at Shanghai Fourth People's Hospital, School of Medicine, Tongji University
Source: Front Microbiol. 2024 Apr 15;15:1361197. doi: 10.3389/fmicb.2024.1361197 (PMC11056568; doi:10.3389/fmicb.2024.1361197)
Supplement: Supplementary file 1 [file Table_1.DOCX]

**Supplementary Figure 1. Flowchart of the patient selection process**

2645 COVID-Omicron BA.2 infected patients from a single-centre study

Excluding patients with age <65(n=807)

1838 elderly patients

Excluding patients with >30% covariate information missing

1608 patients

Excluding patients with a diagnosis of severe disease at admission(n=40)

1568 patients met the criteria above
